# The Effects of Working Memory Load on Auditory Distraction in Adults With Attention Deficit Hyperactivity Disorder

**DOI:** 10.3389/fnhum.2021.771711

**Published:** 2021-11-30

**Authors:** Rina Blomberg, Andrea Johansson Capusan, Carine Signoret, Henrik Danielsson, Jerker Rönnberg

**Affiliations:** ^1^Department of Behavioural Sciences and Learning, Linköping University, Linköping, Sweden; ^2^Swedish Institute for Disability Research, Linköping University, Linköping, Sweden; ^3^Department of Psychiatry, Linköping University, Linköping, Sweden; ^4^Department of Clinical and Experimental Medicine, Linköping University, Linköping, Sweden; ^5^Center for Social and Affective Neuroscience, Linköping University, Linköping, Sweden; ^6^Center for Medical Image Science and Visualization, Linköping University, Linköping, Sweden

**Keywords:** attention deficit hyperactivity disorder, adults, attention, cognitive control, auditory distraction, salience network (SN), working memory, task-based fMRI

## Abstract

Cognitive control provides us with the ability to *inter alia*, regulate the locus of attention and ignore environmental distractions in accordance with our goals. Auditory distraction is a frequently cited symptom in adults with attention deficit hyperactivity disorder (aADHD)–yet few task-based fMRI studies have explored whether deficits in cognitive control (associated with the disorder) impedes on the ability to suppress/compensate for exogenously evoked cortical responses to noise in this population. In the current study, we explored the effects of auditory distraction as function of working memory (WM) load. Participants completed two tasks: an auditory target detection (ATD) task in which the goal was to actively detect salient oddball tones amidst a stream of standard tones in noise, and a visual *n*-back task consisting of 0-, 1-, and 2-back WM conditions whilst concurrently ignoring the same tonal signal from the ATD task. Results indicated that our sample of young aADHD (*n* = 17), compared to typically developed controls (*n* = 17), had difficulty attenuating auditory cortical responses to the task-irrelevant sound when WM demands were high (2-back). Heightened auditory activity to task-irrelevant sound was associated with both poorer WM performance and symptomatic inattentiveness. In the ATD task, we observed a significant increase in functional communications between auditory and salience networks in aADHD. Because performance outcomes were on par with controls for this task, we suggest that this increased functional connectivity in aADHD was likely an adaptive mechanism for suboptimal listening conditions. Taken together, our results indicate that aADHD are more susceptible to noise interference when they are engaged in a primary task. The ability to cope with auditory distraction appears to be related to the WM demands of the task and thus the capacity to deploy cognitive control.

## Introduction

Cognitive control refers to a set of complex cognitive mechanisms that collectively coordinate flexible and goal-directed behavior and include working memory (WM), attention, conflict monitoring, contextual anticipation and inference, inhibition and action selection (Egner, 2017). Importantly, these cognitive mechanisms provide us with the ability to not only selectively prioritize goals/requirements but to also suppress intrusive thoughts, inhibit inappropriate actions and ignore environmental distractions in accordance with those goals/requirements (Badre, 2020). Attention deficit hyperactivity disorder (ADHD) is a neuropsychiatric disorder in which symptoms encompass developmental deficits in cognitive control (Barkley, 1997; Castellanos and Tannock, 2002; van Lieshout et al., 2017; Pievsky and McGrath, 2018). Although once considered a childhood disorder, deficits in cognitive control have been shown to persist into adulthood (Faraone et al., 2015, 2021). In adults, ADHD is heterogenous but some symptoms are more representative of adult-ADHD (aADHD) than others. Symptoms of hyperactivity for instance, have been shown to decrease (or at least manifest in different ways compared to children) whereas attention and WM related difficulties tend to persist into adulthood (Mostert et al., 2015; Uchida et al., 2018). Indeed, deficient WM capacity is one of the most robust associations of impairments in daily functioning in aADHD (Willcutt et al., 2005; Alderson et al., 2013).

Cognitive neuroscience has known for some time that attention modulates sensory processing. Directing attention to a particular sensory modality for instance, can increase cortical activity in primary and secondary processing regions whilst directing attention away from the sensory source can reduce neural activity in said cortical regions. The effect of the latter is considered a mechanism for inhibiting sensory distraction. WM capacity–as an index for more general cognitive control ability–has long been theorized to play a prominent role in constraining distraction. In the auditory domain, empirical studies have shown that individuals with low WM capacity have difficulties hampering the disruptive effects of involuntary orienting to task-irrelevant acoustic stimulation (e.g., Conway et al., 2001; Dige et al., 2010; Sörqvist, 2010a; Hughes et al., 2013; Yurgil and Golob, 2013; Pelletier et al., 2016), although results are not always consistent (e.g., Beaman, 2004; Körner et al., 2017; Nagaraj et al., 2020).

One theory (Sörqvist, 2010b; Sörqvist and Rönnberg, 2014; Sörqvist and Marsh, 2015; Marsh and Campbell, 2016) suggests that WM capacity shields against auditory distraction in two main ways. First, high WM capacity is associated with better cognitive control, thus individuals with high WM capacity are able to maintain a more steadfast locus of attention in the face of challenging demands than their low WM capacity counterparts. Secondly, this uptake in attentional engagement deploys inhibitory mechanisms that suppress neural responses to task irrelevant sounds in accordance with these demands. The effect is reciprocal, in that challenging requirements both increase attentional engagement and decrease susceptibility for distraction within the limits of the individual’s capacity for cognitive control. In support of this theoretical perspective, several studies in typically developed adults (TDa) have shown that increasing WM load in the visual modality results in greater neural attenuation of task-irrelevant auditory stimulation in the brainstem, and auditory cortical processing regions (e.g., Gisselgård et al., 2003; Regenbogen et al., 2012; Sörqvist et al., 2012, 2016).

Heightened auditory distraction is a commonly reported symptom in adults with ADHD (Schulze et al., 2020) and clinically, is associated with more general impairments in attention. Some behavioral studies in adults with ADHD indicate that their susceptibility to auditory distraction is related to a deficient WM capacity (e.g., Dige et al., 2010; Pelletier et al., 2016). However, neuroimaging studies investigating the effects of attentional engagement upon auditory distraction in aADHD are scarce. The main purpose of the current study, therefore, is to investigate whether our sample of aADHD, compared to healthy controls, demonstrate heightened cortical responses to task-irrelevant acoustic stimulation whilst engaged in a visual WM task; and to explore whether cortical attenuation of task-irrelevant acoustic stimulation is associated with individual differences in WM capacity.

To this end, we adopted a similar task paradigm from previous neuroimaging studies in TDa (Regenbogen et al., 2012; Sörqvist et al., 2016) that reported decreases in task-irrelevant auditory processing as a function of visual WM load. In all experimental condition’s participants were exposed to a monotonous tonal signal which included an occasional deviant pitch whilst viewing a sequence of letters in the center of their visual field. In three of four experimental conditions, participants were instructed to ignore the auditory stream and perform one of three *n*-back conditions: 0-, 1-, and 2-back, on the visual letter sequence. In the remaining condition, participants were asked to explicitly attend to the auditory signal and to detect the deviant pitch, i.e., oddball target, whilst ignoring the visual sequence of letters.

fMRI imaging studies in TDa have shown that exogenous responses to task-irrelevant changes in pitch activate auditory core and belt regions as well as the posterior insula (e.g., Mayer et al., 2006; Sabri et al., 2011; Alho et al., 2014, 2015; Huang et al., 2015). These regions are thought to be activated pre-attentively and may generate the early N1 and MMN (mismatch negativity) auditory components in EEG recordings (Edwards et al., 2005; Garrido et al., 2009; Menon and Uddin, 2010; El Karoui et al., 2015; Blenkmann et al., 2018; Citherlet et al., 2020). From previous studies (Gisselgård et al., 2003; Regenbogen et al., 2012; Sörqvist et al., 2012, 2016), we expected that neural responses to the streaming acoustic signal, in these core and belt auditory regions, would attenuate when participants focused their attention on the visual WM task. And we hypothesized that the magnitude of attenuation in the control group, would increase as WM load transitioned from low to high. In contrast, we hypothesized that the ADHD group would show heightened auditory activity under high visual WM load relative to controls and that this heightened activity would negatively correlate with performance on the high-load task. Additionally, based upon findings from Sörqvist et al. (2012) we also expected WM capacity to correlate with auditory attenuation under the high-load condition.

The auditory oddball task was a simple target detection task that was not expected to place demands on WM capacity. The salience network (SN), a network critically involved in cognitive control operations, has been shown to be consistently activated in target detection tasks (Crottaz-Herbette and Menon, 2006). The network is generally thought to be triggered exogenously via communications from sensory processing regions and plays an active role in the vigilant anticipation, detection, and response-mediation of behaviorally salient stimuli (Corbetta et al., 2008; Menon and Uddin, 2010; Cabeza et al., 2012; Vossel et al., 2014). We therefore expected to see increased SN activation in the auditory target detection (ATD) task relative to a resting baseline in both participant groups. Although some resting-state fMRI studies have observed aberrant SN connectivity in ADHD (see Castellanos and Aoki, 2016 for a review), task-based fMRI investigations of the SN in aADHD are rare, particularly in the auditory domain. We therefore additionally tested for between-group differences in auditory–SN connectivity during the ATD task.

## Materials and Methods

### Participants

We recruited two groups of participants: clinically stable adults with ADHD and healthy controls. Inclusion criteria were assessed via a two-step procedure. First, all applicants were required to fill in a digital questionnaire regarding age, health, handedness, alcohol/substance use, diagnosis/es and medications. Applicants were excluded at this stage if they were older than 50 years; were dominantly left-handed; reported having medical or psychiatric conditions or disabilities that could affect the quality of the data (e.g., severe acute psychiatric disorders such as but not exclusively: psychotic disorder, bipolar disorder, current severe MDD; ASD or hearing loss) or reported frequent use of alcohol/substances. Participants medicated with medication that could affect attention or wakefulness such as neuroleptics, sedatives, and/or opioids were excluded. Because a vast majority of aADHD (up to 75%) also have comorbid anxiety and/or depressive symptoms (Kessler et al., 2005; Kooij et al., 2012), ADHD-applicants on stable doses for at least 2 months of common antidepressants were not excluded. Stable medication with SSRI or SNRI indicates stability in the comorbid condition, while not unnecessarily excluding a representative group of participants. In addition, applicants with ADHD were included only if medicating with central stimulants (methylphenidate, dexamphetamine, lisdexamfetamine, etc.) and prepared to undergo a 48 h washout period prior to testing or were currently unmedicated for their ADHD.

Second, inclusion criteria were further assessed on the day of participation. Participants were screened for normal hearing thresholds (<20 dB HL) with pure-tone audiometry at six frequencies ranging from 250 to 8000 Hz (as described in: American Speech-Language-Hearing Association, 2005). Clinical assessments of attention, ADHD-symptom severity, and the presence of comorbid disorders and problems with substance abuse were investigated in more detail via the d2-R Test of Attention (Brickenkamp et al., 2010), the 18-item adult ADHD self-report scale (ASRS) v.1.1 (Kessler et al., 2005; Rodriguez et al., 2007) and the Mini-International Neuropsychiatric Interview (MINI) 7.0.2 DSM-5 for ADHD studies (Sheehan et al., 1998) respectively. Individual scores for the ASRS were calculated by summing scores from items associated with self-reported attentional difficulties (Part A: 1–4; Part B: 1–5), hyperactivity/impulsivity problems (Part A: 5–6; Part B: 6–12), as well as the sum of all 18-items as a general index of symptom severity. Standard scoring procedures were used for each index in the d2-R and MINI.

The data for this study was obtained from 34 participants who met the inclusion and exclusion criteria, forming two equal groups. The ADHD-group consisted of eleven females (age: *M* = 27 years, SD = 7.0) and six males (age: *M* = 29 years, SD = 7.0). 15 out of the 17 ADHD participants were prescribed stimulant medication (eight lisdexamfetamine, six methylphenidate and one dexamphetamine), and abstained from their medication for 48 h prior to testing. The remaining two participants were unmedicated for ADHD. In the ADHD group five participants had SSRI medication and one had lamotrigine in stable doses during at least 2 months. The control group consisted of 13 females (age: *M* = 25 years, SD = 4.9) and 4 males (age: *M* = 26 years, SD = 6.2).

### Materials and Experimental Protocol

The scanning protocol utilized a block design and was programmed in Presentation Software (21.1, build September 05, 2019).^1^ Participants used their right index finger to trigger the response button and response times (RTs), performance accuracy and false alarms (i.e., responding when no target was present) were recorded via a response box (LUMINA, Cedrus Corporation, San Pedro, CA, United States) interfaced with the stimulus presentation. In all experimental blocks, participants were exposed to both auditory and visual stimuli. Auditory stimuli were presented to participants via OptoActive^TM^ active noise canceling headphones (OptoAcoustics Ltd., Tel Aviv, Israel). The noise canceling headphones both passively and actively attenuated the background echo planar imaging (EPI) gradient noise to ∼ 58 dB SPL. And the headphones were kept in place via inflatable positioning pads (Pearltec MRI/CT Multipad Plus, MagMedix, Fitchburg, MA, United States) which also served to minimize head movements within the head cage. Visual stimuli were presented in the center of the visual field via MRI-goggles (Resonance Technology Company, Inc., Los Angeles, CA, United States).

Prior to the scan, subjects underwent a training session to ensure that they clearly understood all the task requirements. For the visual *n*-back task, participants were told to perform one of three *n*-back conditions whilst ignoring a streaming acoustic signal. *N*-back stimuli consisted of a sequence of 15 letters drawn pseudo-randomly from the set: K, M, Q, R, S, T, and W (white text on black background, font size: 18 points). Letters were individually presented for a duration of 500 ms followed by an interstimulus interval indicated by a fixation cross of 1022 ms, and for a total block duration of 22.8 s (equivalent to 30 repetition times; TR = 761 ms). The 0-back condition required participants to press the response button to the target letter, K. In the 1-back condition, the response button was to be triggered when participants detected two consecutive, identical letters. The 2-back condition required participants to respond when they saw a letter identical to a letter presented two trials prior. Each *n*-back block contained four target letters in total.

**FIGURE 1:**
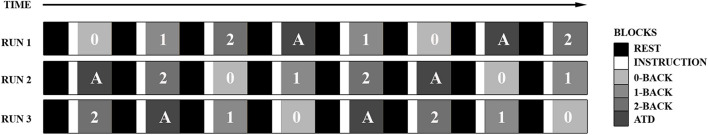
The scanning protocol utilized a block design. Each experimental block (30 TR) was proceeded by a resting baseline condition (20 TR) and every experimental block was initiated by task instruction (7 TR). The figure depicts the presentation order of the experimental conditions which were partially counterbalanced over the entire experiment and divided into three runs of eight task blocks (3 × 456 TR). Each experimental condition was presented twice per run.

In the ATD task, participants were told to ignore the visual *n*-back stimuli and instead shift their attention to the streaming auditory stimulus. The task required participants to press the response button every time they heard a deviant tone (1000 Hz) amidst a stream of standard tones (500 Hz). Tones of 150 ms (rise and fall = 22 ms) were presented every 104 ms with a total of four deviant tones within random distances of circa 2–6 s per 22.8 s experimental block. The perceived loudness of the standard and deviant tones was set at −16 LUFS and presented to participants at a sound pressure level of ∼ 75 dB. The presentation order of the experimental conditions was partially counterbalanced over the entire experiment and divided into three runs of eight task blocks, wherein each experimental condition was presented twice per run (see Figure 1). Every experimental block was proceeded by a 15.2 s resting baseline condition (=20 TR) and initiated by a 5.3 s (=7 TR) task instruction.

Outside of the scanner, and in a quiet room, participants completed two WM span tests. The Reading span task (Rönnberg et al., 1989) to measure WM maintenance, and the Size-comparison span test (Sörqvist et al., 2010) as a measure of both WM gating and maintenance; the procedures for which are published in Blomberg et al. (2019).

### Image Acquisition

Whole-head fMRI scans were performed on a Siemens Prisma 3T scanner with a 64-channel head coil at the Centre for Medical Imaging and Visualization (CMIV), Linköping University Hospital, Sweden. A 3D, T1-weighted MPRAGE (magnetization prepared rapid gradient echo) anatomical scan was acquired with the following parameters: repetition time (TR) = 2300 ms; echo time (TE) = 2.36 ms; flip angle (FA) = 8°; field of view (FOV) = 250 × 250 × 225 mm; acquisition matrix = 288 × 288 × 208; slice orientation = sagittal; slice thickness = 0.9 mm; number of slices = 208; voxel size = 0.87 × 0.68 × 0.9 mm. Whole brain, blood oxygen level dependent (BOLD) fMRI was conducted using EPI with the following parameters: TR = 761 ms; TE = 24 ms; FA = 53°; FOV = 204 × 204 mm; acquisition matrix = 68 × 68; number of slices = 45; slice thickness = 3 mm; voxel size = 3 × 3 × 3 mm. Field map imaging was performed with a double-echo spoiled gradient echo sequence, generating one magnitude and two phase images [TR = 520 ms; TE = 4.92/7.38 ms; FA = 60°; total EPI readout time = 16.415 ms; blip direction = 1]. Participants underwent a 12 min resting state scan (this data is reported elsewhere) immediately prior to the current study’s task-based scan. Before commencing the task-based session, participants were removed from the scanner so that the ear plugs they wore during the resting state scan could be removed and so that the active noise-canceling headphones could be refitted and recalibrated to ensure the quality of the signal-to-noise ratio.

### Pre-processing

The CONN toolbox v.20.b (Whitfield-Gabrieli and Nieto-Castanon, 2012, 2017; RRID:SCR_009550)^2^, which is powered by SPM12 (Statistical Parametric Mapping v. 12, Wellcome Department of Imaging Neuroscience, University College London, United Kingdom), was used to pre-process the data in MATLAB R2020B software. We used the default pre-processing pipeline for volume-based analyses but with indirect normalization to standard stereotactic (MNI) space as we had obtained gradient field maps during image acquisition (Nieto-Castanon, 2020). The pipeline consisted of the following five steps and parameters:

(1) Functional realignment and unwarp with the use of fieldmaps for susceptibility distortion correction.
(2) Slice-timing correction wherein the predefined “Siemens interleaved” acquisition sequence was selected from the CONN toolbox user interface.
(3) Outlier identification in which framewise displacements greater than 0.9 mm or global BOLD signal changes above five SD were flagged as potential outliers.
(4) Indirect segmentation and normalization in which the functional data was first co-registered to the anatomical data using SPM12 inter-modality co-registration procedure with a normalized mutual cost function (Collignon et al., 1995; Studholme et al., 1998). Second, the anatomical data was normalized into standard MNI-space and segmented into gray matter, cerebrospinal fluid, and white matter tissue classes using SPM12 unified segmentation and normalization procedure (Ashburner and Friston, 2005) with the T1-weighted volume as a reference image. Third, the same estimates of the deformation field from the unified segmentation and normalization procedure on the anatomical data was applied to the functional data. CONN toolbox’s default probability tissue maps were selected: 180 × 216 × 180 mm bounding box with 1 and 2 mm isotropic voxels for the anatomical and functional data respectively.
(5) The functional data was smoothed using spatial convolution with CONN toolbox’s default Gaussian kernel recommendation of 8 mm FWHM (full width half maximum).

### Regions of Interest Definitions

#### Auditory Regions of Interests

We selected four anatomical regions of interests (ROIs) within each hemisphere that have been consistently associated with the early detection of changes in frequency/pitch; these included Heschl’s gyrus (HG) of the auditory core; the planum polare (PP) and planum temporale (PT) of the auditory belt; and the posterior insula (Bamiou et al., 2003; Moerel et al., 2014). The Harvard-Oxford structural atlas implemented in the CONN toolbox was used to define the HG, PP, and PT ROIs. Because the insula is a relatively large structure, we used the SPM anatomy toolbox 3.0 (Eickhoff et al., 2005, 2006, 2007) to generate two posterior histological subdivisions: a dysgranular ROI (dpI) just posterior to the central sulcus comprising of Id2 and Id5; and a granular posterior ROI (gpI) comprising of Ig1, Ig2, and Ig3. In total, we investigated 10 auditory ROIs (five in each hemisphere).

#### Salience Network Regions of Interests

The CONN-toolbox’s network atlas was used to select SN-hub ROIs with predefined MNI centroids (*x*, *y*, *z*) consisting of the: dorsal anterior cingulate (dACC; 0, 22, 35); left (−44, 13, 1) and right (47, 14, 0) anterior insula (aI); left (−32, 45, 27) and right (32, 46, 27) rostral prefrontal cortex (rPFC); and the left (−60, −39, 31) and right (62, −35, 32) supramarginal gyrus (SMG).

### Statistical Analysis

#### Group Descriptives and Behavioral Analysis

One-way ANOVAs were used to test differences in means between groups, except when the assumption of equal variances was violated in which a Welch-test was applied. A response on one of the ASRS items was missing for one of the control participants on the hyperactivity/impulsivity scale, so this participant was excluded from the *F*-test of group differences in hyperactivity/impulsivity. Also, one of the ADHD-participants’ behavioral results from the scanner task was lost due to a technical error. Statistical comparisons of fMRI task performance (accuracy, RT, and false-alarms) were therefore conducted on 16 of the 17 ADHD participants. The RTs we report are based on accurate trials only.

#### Univariate Activation Analysis

The univariate activation analysis was conducted in the SPM12 analysis software. The pre-processed functional data for each participant was entered into a general linear model (GLM) that included for each run; five regressors representing the four experimental conditions and the resting baseline condition. Six motion parameters (obtained from the realignment procedure during pre-processing) were included as covariates of no interest. The model additionally included three regressors representing the mean signal across the three runs. SPM12’s default high-pass filter of 128 s was applied prior to parameter estimation to control for low-frequency signal confounds. Contrast estimates for the effects of ATD (ATD--rest), and the change in auditory activity from attending to the auditory modality and attending to the visual modality for each WM load condition (0-back--ATD, 1-back--ATD, and 2-back--ATD) were extracted for each participant by way of the SPM-compatible REX (ROI Extraction) tool^3^; and then used for group-level ROI analyses in IBM SPSS v.27 statistical analysis software. Because we did not have specific hypotheses of interactions between hemispheres, auditory ROI analyses were performed per hemisphere, which also allowed us to preserve statistical power. Differences between hemispheres are thus only qualitatively assessed in the results.

Independent *t*-tests were used to explore group-level SN, and auditory ROI BOLD activation for the ATD task and resulting *p*-values were Bonferroni corrected for multiple comparisons. Two-tailed Spearman’s correlation analysis was also performed to determine if performance on the ATD task correlated with activity in any of the auditory/SN ROIs. For the visual WM task, a mixed repeated measures ANOVA (one per hemisphere) was used primarily to analyze the main effect of WM load and the WM load × Group interaction; and also, to determine whether there was a linear relationship between auditory ROI attenuation and visual WM load. Greenhouse-Geisser correction of degrees of freedom was applied when the sphericity assumption was violated, and *p*-values from relevant *post hoc* tests were Bonferroni corrected. Two-tailed Spearman’s correlation analysis was used to test the hypothesis that WM capacity would positively correlate with auditory attenuation under high cognitive load. We additionally explored whether the strength of auditory attenuation in the high-load condition was correlated with improved task performance and individual differences in self-rated inattentiveness (derived from the ASRS, see Table 1).

**TABLE 1:** Group descriptives for education, hearing acuity and neuropsychological assessments of ADHD-related symptomology.

	**Controls (*N* = 17)**	**ADHD (*N* = 17)**
**Current/highest education level**	***N***	***N***

Upper secondary	1	4
Undergraduate	14	13
Post-graduate	2	–

**Hearing acuity (dB)**	***M* (SD)**	***M* (SD)**

Pure-tone average	−2.5 (4.1)	−0.3 (7.4)

**d2-Test Attention (standard score)**	***M* (SD)**	***M* (SD)**

Concentration	103 (7.1)	100 (9.0)
Processing speed	103 (15.6)	106 (15.2)
Precision	100 (9.9)	97 (11.6)

**ASRS v.1.1 (aggregate score)**	***M* (SD)**	***M* (SD)**

Inattention	12.8 (4.7)	27.5 (3.9)
Hyperactivity/Impulsivity	11.9^†^ (5.7)	23.0 (8.1)
Total ASRS	24.6^†^ (9.2)	50.5 (11.5)

**MINI 7.0.1 for ADHD studies**	***N***	***N***

ADHD: Inattentive	1	6
ADHD: Impulsive/Hyperactive	–	–
ADHD: Combined	–	11

*^†^Mean derived from 16 out of the 17 Controls as item 8 (Part B) was missing for one of the participants.*

Although not associated with the main hypotheses of this study, for completeness we additionally conducted a voxel-wise whole brain analysis. Group level, one-sample (within-groups) and two-sample (between-groups) *t*-tests were performed on the four contrasts of interest of which both ADHD > Controls and Controls > ADHD comparisons were explored. Resulting statistical maps were family wise error (FWE) corrected using *p* < 0.05 with a minimum extent threshold of *k* = 10 voxels.

#### Functional Connectivity Analysis

An additional denoising procedure was performed on the pre-processed data) using the CONN-toolbox’s default denoising pipeline (Whitfield-Gabrieli and Nieto-Castanon, 2012; Nieto-Castanon, 2020). The pipeline consisted of the following two steps:

(1) Nuisance covariates derived from CONN-toolbox’s implementation of anatomical component-based correction (aCompCor) were entered into an ordinary least squares regression in order to remove confounding effects on the estimated BOLD signal in each voxel per subject and run. The covariates included five noise components from cerebral white matter; five noise components from cerebrospinal areas; 12 subject motion components (three translation, three rotation and their first-order temporal derivatives), outlier scans identified in the pre-processing procedure (see section “Pre-processing,” step 3) and components representing the effect of each task-condition convolved with the canonical hemodynamic response function in order to reduce the influence of slow trends, initial magnetization transients as well as constant task-related effects.
(2) Temporal band pass filtering (high pass: 0.008 Hz, low pass: 0.09 Hz) on the BOLD signal was applied in order to minimize the influence of physiological head motion and other noise sources.

First, and second level functional connectivity analysis was further conducted in the CONN-toolbox. Each participant’s denoised, voxel-wise BOLD time series data (concatenated over runs) was averaged within each auditory, and SN ROI per experimental condition. Then, for each task condition, first-level HRF-weighted ROI–ROI connectivity analyses were performed in which the correlation coefficient of each ROI to all other ROIs was calculated. Resulting correlation coefficients were Fisher *z*-transformed. Each participant’s ROI-ROI connectivity matrices were then entered into a second level GLM to obtain group-level estimates for the ATD condition. Univariate parametric statistics were used to perform connection-based, between-group inferences (ADHD > Controls) across all pairs of ROIs. The connection-level significance threshold of *p* < 0.05 was conservatively corrected by way of FDR-correction (false discovery rate).

## Results

### Group Descriptives

Table 1 presents group descriptives for education, hearing acuity, MINI, ASRS and d2-R measures. The majority of participants (both ADHD and Controls) were studying (or had completed studies) at an undergraduate level of education. There were no differences between groups in age *F*(1, 33) = 0.35, *p* = 0.557; or hearing acuity, *Welch’s F*(1, 25.3) = 1.4, *p* = 0.252. The majority of individuals in the control group reported having relatively infrequent difficulties with inattention and hyperactivity/impulsivity as measured by the ASRS and MINI. Although one control participant was categorized as inattentive on the MINI, this participant only scored 17 for inattentiveness on the ASRS which is well under the cut-off score (24) for diagnostic evaluation. However, excluding this participant from the analysis did not change the pattern of results. The reason for this discrepancy between scales may be because the MINI requires a forced choice, yes/no answer for each item, whereas the ASRS is more nuanced and allows the participant to reflect and grade in more detail the frequency of symptoms. The majority of ADHD participants (11) had the combined subtype, the remaining six had mainly inattentive subtype according to MINI. In addition, our sample of aADHD had, as expected, significantly higher ASRS scores on both inattentive *F*(1, 32) = 97.1, *p* < 0.000, η^2^ = 0.75; and hyperactivity/impulsivity *F*(1, 31) = 20.6, *p* < 0.000, η^2^ = 0.40; subscales compared to Controls. The ADHD group did not however demonstrate significantly poorer performance than Controls on the d2-Test measures of concentration *Welch’s F*(1, 30.4) = 1.3, *p* = 0.262; processing speed *F*(1, 32) = 0.31, *p* = 0.581; or precision *F*(1, 32) = 0.63, *p* = 0.432.

### Behavioral Results

Statistical details of group differences in WM capacity and in-scanner performance measures are reported in Table 2. In measures of WM capacity, our sample of aADHD performed on par with Controls. In the *n*-back task, no differences in mean RTs, or the number of false alarms were observed between groups. Group means in accuracy performance were slightly lower in the ADHD group compared to Controls, but the difference in means was only significant in the highest load (2-back) condition (η^2^ = 0.303). We were surprised to see relatively low accuracy scores on the ATD task from both groups. Closer inspection of the data revealed that the majority of errors were associated with false alarms and occurring milliseconds–seconds prior to the onset of the oddball. Thus, the low accuracy scores in the ATD task was likely an outcome of participants responding impulsively to the anticipation of an oddball (see section “Auditory Target Detection in Noise” in the Discussion).

**TABLE 2:** Descriptives and one-way ANOVA results (Welch test was applied when the assumption of homogeneity was violated) for group comparisons of working memory (WM) capacity and in-scanner task performance on the *n*-back and auditory target detection (ATD) conditions. RT: response times in milliseconds and correspond to accurate trials only. Due to a technical error, fMRI-task performance data was missing for one ADHD participant.

		**Controls *M* (SD)**	**ADHD *M* (SD)**			
**WM capacity**		***N* = 17**	***N* = 17**	***F***	***df***	***p***

Reading span		0.51 (0.12)	0.46 (0.16)	1.29	1, 32	0.265
Size comparison span		0.71 (0.12)	0.63 (0.23)	1.35	1, 24.8	0.256

**fMRI-task performance**		***N* = 17**	***N* = 16**	***F***	***df***	***p***

RT	0-back	375 (38)	390 (57)	0.71	1, 31	0.405
	1-back	462 (83)	503 (71)	2.28	1, 31	0.141
	2-back	525 (119)	534 (105)	0.06	1, 31	0.814
	ATD	283 (31)	296 (44)	0.87	1, 31	0.358
Accuracy	0-back	0.99 (0.02)	0.95 (0.13)	1.57	1, 31	0.219
	1-back	0.93 (0.10)	0.86 (0.17)	2.33	1, 31	0.137
	2-back	0.89 (0.06)	0.74 (0.16)	13.45	1, 31	0.001
	ATD	0.87 (0.07)	0.84 (0.08)	0.91	1, 31	0.349
False alarms	0-back	0.2 (0.6)	0.6 (0.8)	2.62	1, 31	0.116
	1-back	1.2 (1.8)	2.7 (2.8)	3.36	1, 31	0.077
	2-back	3.2 (2.0)	4.3 (3.3)	1.32	1, 31	0.259
	ATD	3.1 (1.8)	3.6 (2.0)	0.91	1, 31	0.349

### Whole Brain Analysis

Two-sample *t*-tests of whole brain voxel-wise activity did not detect significant differences between groups in any contrasted condition after controlling for multiple comparisons. Within-group analyses however did reveal significant cluster-level activations in all contrast conditions. Cluster-level statistics and slice-by-slice maps of significant effects for within-groups analyses are reported in Supplementary Table 1 and Supplementary Figures 1–4. For the ATD–rest contrast, activity was observed in auditory, superior temporal and supplementary motor cortices within both groups. For the *n*-back–ATD contrasts, the number of active regions increased with increasing WM load and was observed mainly in higher order visual processing regions, the exterior cerebellum as well as superior partial and middle frontal gyri in both groups.

### Auditory Target Detection

**FIGURE 2:**
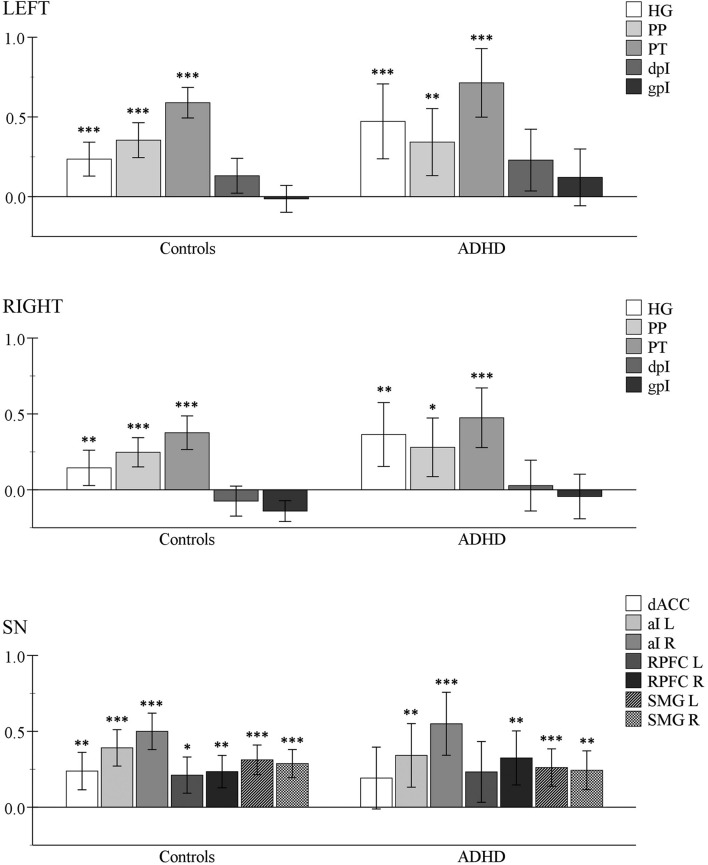
Within-group analysis results for the auditory target detection contrast (ATD > rest). One-sample *t-*tests were used to determine if the mean BOLD activity within each ROI (per group) was greater than zero. *Y*-axis corresponds to mean beta image values. Asterisks indicate Bonferroni-corrected levels of significance: ^∗^*p* < 0.05, ^∗∗^*p* < 0.01, ^∗∗∗^*p* < 0.001; error bars = 95% CI. Top panel: left auditory ROIs. Middle panel: right auditory ROIs. Bottom panel: salient network (SN) hub-regions. HG, Heschl’s gyrus; PP, planum polare, PT, planum temporale, dpI, dysgranular posterior insula; gpI, granular posterior insula; dACC, dorsal anterior cingulate cortex; aI, anterior insula; RPFC, rostral prefrontal cortex; SMG, supramarginal gyrus; L, left; R, right.

Figure 2 presents the detailed results of the univariate ROI analysis for the ATD task (see also Supplementary Table 2). Significant increases relative to the resting baseline for the ATD condition were observed in all SN-hubs in Controls, and all except the dACC and left rPFC in ADHD when correcting for multiple comparisons (i.e., all seven hub ROIs) but uncorrected *p*-values were significant. Between-group comparisons revealed no significant differences for the SN-rest contrast suggesting that all seven SN-hubs were on average, more active relative to baseline in both groups. Initial analysis of the auditory ROIS indicated that only the core, and belt ROIs were significantly more activated than baseline during target detection. Hence, contrary to expectations from previous literature, our posterior insula ROIs were not actively involved in the ATD task; and this was evident in both groups. However, we considered that this may be a result of averaging over relatively large regions of the posterior insula and that a within ROI cluster analysis may prove more informative. To investigate, we used the SPM wfu_pickatlas tool to perform cluster analyses. Results identified significantly active clusters of 26 voxels (*p* FWE-corr = 0.024) in the left dpI (peak coordinates: −46, −8, 6) and eight voxels (*p* FWE-corr = 0.035) in the gpI (peak coordinates: −34, −30, 8) in the ADHD group. In the Control group, a significant cluster of 38 voxels (*p* FWE-corr = 0.011) was identified in the left dpI (peak coordinates: −40, −2, 2). Thus, in line with previous studies implicating the posterior insula in early pitch discrimination tasks, our data does indicate that at least the left posterior insula was involved in our ATD task. Statistical between-group comparisons did not reveal any differences in auditory cortical activation, indicating that all auditory ROIs were similarly activated in both groups throughout the ATD task.

**FIGURE 3:**
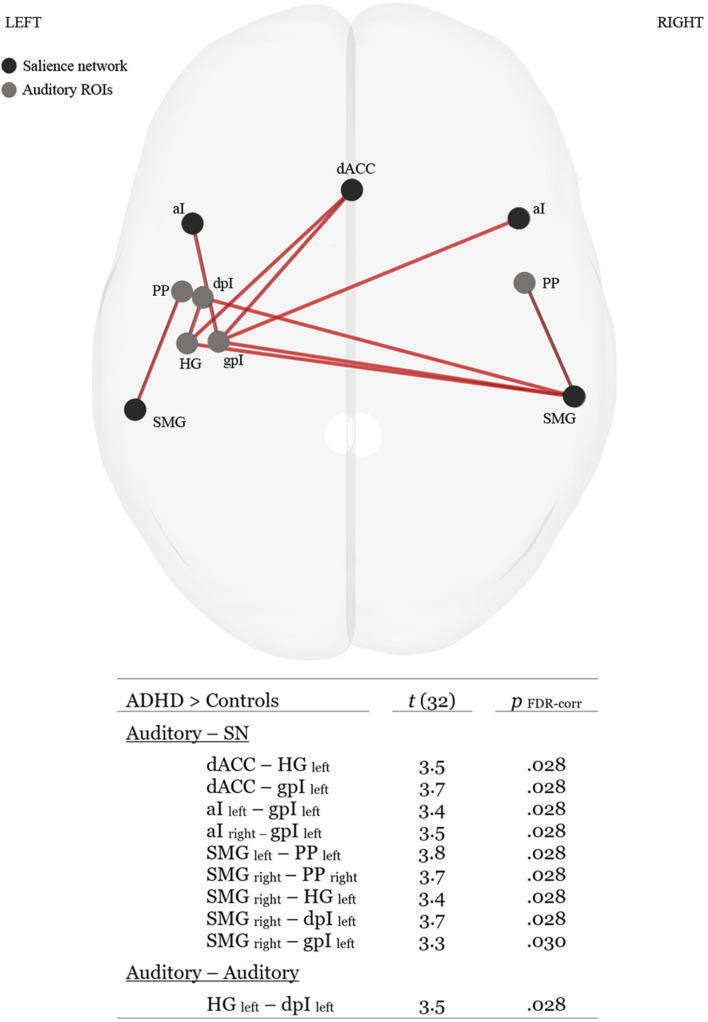
Superior glass-brain perspective and tabulated statistics of ROI–ROI connections that were significantly stronger (*p*_*FDR–corr*_ < 0.05) for the ADHD group in the ATD task. HG, Heschl’s gyrus; PP, planum polare, dpI, dysgranular posterior insula; gpI, granular posterior insula; dACC, dorsal anterior cingulate cortex; aI, anterior insula; SMG, supramarginal gyrus.

Functional connectivity analysis revealed group differences in ROI–ROI connectivity for the ATD task. ADHD participants demonstrated stronger auditory-SN connectivity than Controls of which the majority of connections involved left lateralized auditory communications with dACC, aI and SMG hubs of the SN (see Figure 3 for details). We additionally investigated if individual differences in behavioral performance on the ATD task would correlate with activity levels in any of the auditory or SN ROIs. Here we observed a negative correlation between RTs associated with accurate trials and increases in BOLD activity the right SMG, *r* = −0.39, *p* = 0.027. Hence, faster RTs were associated with heightened activity of the right SMG. No other significant brain–performance correlations were observed.

### Effect of Working Memory Load on Auditory Attenuation

**FIGURE 4:**
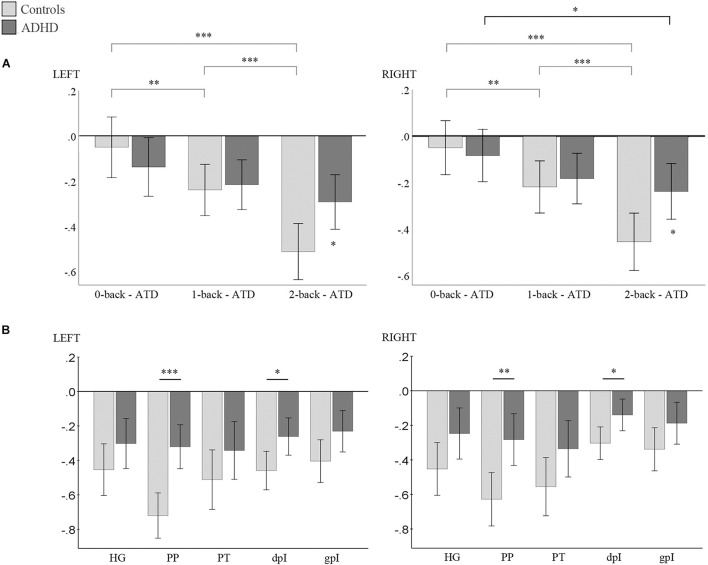
**(A)** Results of *post hoc* analysis for the WM load × Group interaction. Within group differences indicated by horizontal lines above graph. Between group differences indicated by asterisk above error bars. **(B)** Results of *post hoc* analysis for the ROI × Group × High load interaction. Between-group differences indicated by horizontal lines above graph. Left panel: left hemisphere; right panel: right hemisphere. Bonferroni correct *p*-values: ^∗^*p* < 0.05, ^∗∗^*p* < 0.01, *^∗∗∗^p* < 0.001; error bars = 95% CI. ATD = auditory target detection. *y*-axis = estimated marginal means. HG, Heschl’s gyrus; PP, planum polare, PT, planum temporale, dpI, dysgranular posterior insula; gpI, granular posterior insula.

Results of the mixed repeated-measures ANOVAs are displayed in Figure 4. Both hemispheres presented evidence for a main effect of auditory attenuation as a function of WM load: *F*_left_ (2, 64) = 34.6, *p* = 0.000; *F*_right_ (2, 64) = 29.3, *p* = 0.000); and within-participant contrasts confirmed that the effect was linear: *F*_left_ (1, 32) = 30.9, *p* = 0.000; *F*_right_ (1, 32) = 45.6, *p* = 0.000). The interaction term, WM load × Group, was also significant in both hemispheres: *F*_left_ (2, 64) = 8.8, *p* = 0.000; *F*_right_ (2, 64) = 6.2, *p* = 0.003, and *post hoc* inspection of the interaction indicated that the Control group’s auditory responses to the task-irrelevant sound source decreased significantly as WM load increased, and was more suppressed compared to ADHD participants in both hemispheres during the high-load condition (see Figure 4A and Supplementary Table 3 for *post hoc* results). Auditory attenuation increased marginally in the ADHD group across load conditions and was shown to be statistically significant only in the right hemisphere between the 0- and 2-back conditions.

A significant, main effect of ROI was also observed in both hemispheres: *F*_left_ (2.5, 78.9) = 13.2, *p* = 0.000; *F*_right_ (2.1, 67.7) = 14.5, *p* = 0.000).; and the interaction term ROIs × Group was consequently significant: *F*_left_ (4.128) = 5.6, *p* = 0.000; *F*_right_ (4.128) = 3.7, *p* = 0.007. The interactions: WM load × ROI, and WM load × ROI × Group were not significant in either hemisphere, nonetheless *post hoc* analysis of the WM load × ROI × Group interaction was conducted as we consider it necessary to report the specific ROIs where group differences in attenuation were observed. In both hemispheres, attenuation was significantly less evident in the PP and the dpI for the ADHD group compared to Controls under the high-load condition (see Figure 4B and Supplementary Table 3 for detailed results). Trends in the remaining ROIs were observable but the *post hoc* test did not reach the conservatively corrected threshold for significance.

**FIGURE 5:**
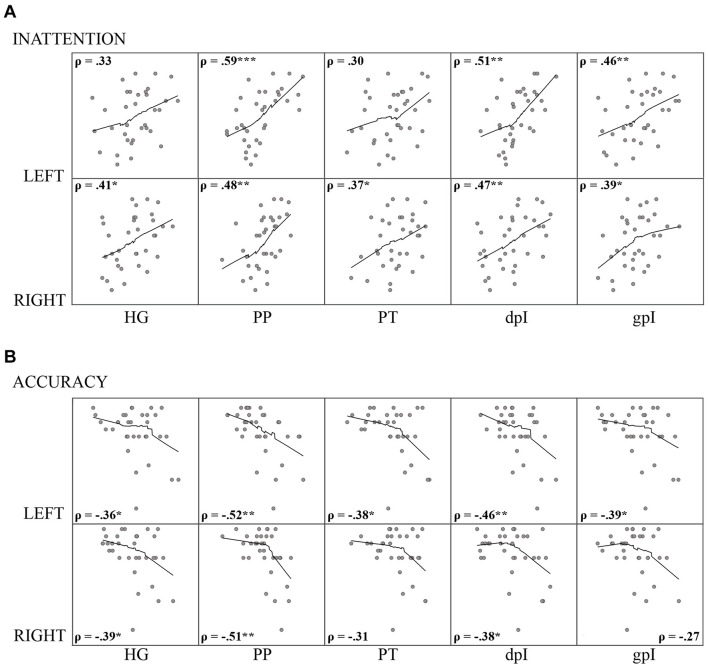
Results of auditory ROI Spearman’s rho (ρ) correlation analysis between the mean change in attenuation under high visual working memory load (2-back–ATD) and **(A)** self-rated inattention; and **(B)** 2-back task accuracy performance. Upper and lower rows of scatter plots correspond to left and right hemispheres, respectively. Asterisks indicate the correlation was significant (two-tailed) at thresholds: *p < 0.05; **p < 0.01; *** *p* < 0.001. Loess trend lines were approximated from a data span of 0.75 with a gaussian kernel. HG, Heschl’s gyrus; PP, planum polare, PT, planum temporale, dpI, dysgranular posterior insula; gpI, granular posterior insula.

We correlated the mean change in auditory attenuation under the high-load condition within each ROI with our WM capacity measures: Reading span and SIC-span. Contrary to expectations, we did not observe a significant relationship with WM capacity in any auditory ROIs. However, the magnitude of auditory attenuation in the high-load condition did correlate significantly with individual differences in self-rated inattentiveness across the majority of auditory ROIs (see Figure 5A for details) which suggests that participants who have difficulties suppressing exogenously triggered responses to task-irrelevant auditory stimulation are also highly susceptible to distraction. In addition, accuracy performance on the 2-back condition also correlated with attenuation levels in the majority of auditory ROIs (see Figure 5B). No other performance related correlations were observed. Taken together, these findings support the hypothesis that heightened cortical responses to task-irrelevant auditory stimulation can both negatively impact task performance and contribute to subjective experiences of auditory distraction.

## Discussion

The main purpose of the current study was to investigate how attentional engagement impacts exogenous cortical responses to acoustic stimulation, and whether adults with ADHD (aADHD) have difficulties hampering auditory distraction. As far as we can determine, we are the first fMRI study to demonstrate that aADHD have difficulty hampering early auditory cortical responses to task-irrelevant sound when required to focus on a cognitively demanding task. When participants’ attention was focused on the auditory modality, auditory cortical activity was enhanced relative to a resting baseline; and when attention was directed away from the auditory modality and toward a visual WM task, auditory processing was attenuated. The degree of attenuation in auditory regions was relative to cognitive load demands, and by extension, endogenous attentional engagement toward the visual task. Importantly, the relationship between attentional engagement and auditory attenuation proved less efficient in aADHD than our matched sample (age and gender) of TDa. In particular, aADHD were had heightened cortical responses to task-irrelevant auditory stimulation and poorer performance capacity in the most challenging WM condition. In addition, although aADHD performed on par with TDa in the ATD task, functional communications between the SN and auditory ROIs were stronger in aADHD. Taken together, the results indicate that aADHD are more susceptible to noise interference when they are engaged in a primary task. How well they cope with noise interference appears to be related to the WM capacity demands of the task. We discuss these results in more detail in the proceeding subsections.

### Working Memory Capacity and Auditory Distraction

We were not expecting aADHD to perform on par with Controls in measures of WM capacity. In a previous study performed by our lab group (Blomberg et al., 2019), adolescents (<18 years) with ADHD demonstrated significantly reduced capacity than matched (age and gender) healthy controls on both the Reading span and the Size-comparison span tasks. This finding, in combination with general associations of ADHD with deficient WM capacity (Willcutt et al., 2005; Alderson et al., 2013) was a major premise for using these measures again in our adult sample. Although ADHD is known to persist into adulthood in over half of patients, numerous researchers have noted that many neurocognitive deficits normalize after the developmental transition into adulthood and suggest that ADHD may mainly be attributable to a developmental delay (Shaw et al., 2007; de Zeeuw and Durston, 2017). A related, yet different perspective, associates ADHD with life-time subcortical (basal ganglia and cerebellum) dysfunction, and that age-related reductions in neurocognitive deficits in some cases are attributable to a healthy maturation of frontal lobes and the improved ability to issue cognitive control over subcortical systems (Halperin and Schulz, 2006). Presumably, such developmental differences between groups were exemplified through our WM capacity measures in our previous study with adolescents (Blomberg et al., 2019). We similarly note that concentration scores from the d2-Test of attention were also differential between groups in our adolescent study, but not in the current study. Because our ADHD participants consisted mostly of young adults undergoing higher education and performed on par with Controls in WM and the d2-Test, it is tempting to consider our sample as relatively “high functioning”–a notion that fits well with the aforementioned developmental models of ADHD. However, we must take into consideration that our ADHD group had poorer accuracy performance in the demanding, in-scanner WM condition (2-back). Hence, there is some evidence to suggest that some WM related difficulties reside in our sample of young adults.

Possibly, the neurocognitive profile of ADHD in adults may be more readily observable under challenging experimental contexts. Our sample of aADHD may well have been able to maintain a commensurate number of items in WM to that of TDa in a quiet, isolated room when undergoing the Reading span and Size-comparison span tests; but during the scanning session, the addition of irrelevant sound stimuli and background noise whilst performing the *n*-back task likely rendered the ADHD group vulnerable to cognitive interference. The latter conclusion is consistent with two other studies in aADHD. Pelletier et al. (2016) reported that visual serial recall performance in their sample of aADHD was more strongly affected by the presence of irrelevant sound; and Dige et al. (2010) observed greater noise interference effects upon verbal memory in a dichotic memory task relative to controls.

We had hypothesized that the additional demands placed by the acoustic environment in the scanner would impact cognitive processing, particularly under the high-load condition, given that control resources needed to be shared between both regulating attention and WM toward one modality whilst suppressing exogenously evoked distractions in another. On this basis, we also expected that participants with high WM capacity would demonstrate more efficient neural attenuation of the acoustic environment and perform overall better on the 2-back condition, a hypothesis supported in previous work by Sörqvist et al. (2012) from our work group (see Sörqvist and Rönnberg, 2014 for a review). This was not supported in our results. We note that in Sörqvist et al. (2012), the authors derived a composite measure of WM capacity from several complex span tasks and the composite score only correlated with auditory brainstem attenuation to task-irrelevant sound in their 3-back condition (i.e., not 2-back). It is thus possible that the WM capacity measures we used in the current study were not sufficiently sensitive indexes of the type of control mechanisms involved in the successful regulation of attention in our 2-back condition.

We did however observe a relatively robust negative correlation between individual differences in auditory attenuation of task-irrelevant sound and 2-back task accuracy. This result provides us with an indication that better cognitive performance on the high-load WM task was intricately related to participants’ capacity to regulate resources between modalities and inhibit distraction. In additional support of this conclusion, participants’ subjective experiences of inattentiveness in daily life (as determined by the ASRS) also robustly correlated with the degree of auditory cortical attenuation under the high-load condition. Our participants with ADHD, that consisted of inattentive or combined (i.e., both inattentive and hyperactive/impulsive) subtypes, were thus more susceptible to auditory distraction and demonstrated poorer performance capacity under the most challenging WM condition.

### Auditory Target Detection in Noise

The ATD task, in which an infrequent “oddball” tone was to be detected amongst a rapid stream of standard tones, places relatively little demand upon cognitive control systems when performed in quiet and should result in high accuracy scores–especially in TDa with normal hearing thresholds. Despite our use of an active noise canceling system which enabled us to present the acoustic stimulus at an audible signal-to-noise ratio of ∼17 dB SPL, behavioral responses from both groups contained a relatively high number of false alarms that occurred just milliseconds-to-seconds prior to the onset of a target. This result suggests that participants were likely responding impulsively to the anticipation of an oddball. The presentation times of oddball-targets were randomly distributed over the 22 s duration of each task-block (four targets per block), so anticipating the exact timing of a target was not possible; however, the audibility of background scanner noise may have been interfering with expectations of an oddball occurring, resulting in impulsive false-alarm responding.

We expected the SN to be heavily involved in the ATD task due to its involvement in vigilant anticipation, detection, and response-mediation of behaviorally salient stimuli; and we explored this network on the grounds that a number of studies have reported aberrant SN functional connectivity in ADHD (see Castellanos and Aoki, 2016 for a review). Significant hyper auditory–SN connectivity was observed in aADHD. Although our test protocol makes it difficult to discern the exact reason for these stronger SN–auditory interactions in the ADHD group, the explanation we consider aligns well with our more general thesis that noise places increased demands on control systems. Given their reported difficulties with inattentiveness, the suboptimal listening situation may have impacted ADHD participants such that the informational exchange between auditory ROIs and SN-hubs was enhanced to facilitate successful oddball detection. As we observed in the 2-back task, heightened auditory responses to the acoustic environment were associated with poorer accuracy performance and symptomatic inattentiveness. And we argued that the challenging 2-back condition taxed cognitive resources and impeded on ADHD participants’ ability to suppress exogenous responses to the acoustic environment. Even though we observed differences in functional connectivity between groups, there were no differences in performance outcomes on the ATD task. Because we do not consider the ATD task cognitively challenging, it is likely our sample of aADHD had the resources available to enhance oddball detection and reduce erroneous responding under suboptimal listening conditions; hence compensating for their presumed symptomatic susceptibility to noise interference.

Increased activity in the right SMG of the SN was also negatively correlated with individual differences in RTs on the ATD task, of which the RTs corresponded only to accurate trials. Hence, faster, accurate RTs were associated with increased activity in the right SMG. The SMG is a core hub of the SN, and the right lateralized region in particular has been implicated in the mediation of exogenous attention to behaviorally salient events (Corbetta and Shulman, 2002; Corbetta et al., 2008; Cabeza et al., 2012; Vossel et al., 2014). Given this proposed functional role of the right SMG in sensory target detection, our combined results suggests that rapid and successful detection of the oddball was associated with effective assignment of saliency to the oddball tone mediated by the right SMG.

### Clinical Implications

With respect to our results, it appears that “high-functioning” aADHD perform relatively well during cognitive tasks but at a greater cost. This notion is in line with earlier literature indicating that a college student with ADHD may need to work twice as hard as their non-ADHD counterparts in order to achieve satisfactory grades (Faraone et al., 2015). Hence, in order to perform in the complex reality of a college or work environment, aADHD may need to deploy more cognitive effort in order to inhibit distraction. The implication of this increased effort is potentially a contributing factor to the reportedly higher levels of perceived stress in college students with ADHD symptoms and the prevalence of anxiety and stress-related pathologies in aADHD (Salla et al., 2019; Gbessemehlan et al., 2020; Öster et al., 2020). Given that some participants were unmedicated during this study, we suggest that more research is needed to understand how medication can compensate for these deficiencies. To prevent stress related problems later in life, clinical interventions should also address adapting work/study environments in order to minimize disturbance and utilize psychoeducation to better improve stress and time-management.

### Limitations

This study has several limitations. First, comparisons of ROIs and networks are difficult across literature due to differences in nomenclature and methods of definition. Hence, our decision for using predefined ROIs and networks through freely available analysis applications, together with as many default analysis settings as possible, was in the hope of facilitating future researchers’ ability to confer/replicate our results. Second, scanner noise was audible throughout the entire experiment. Even during the resting baseline condition. Although we were able to present the auditory stimulus at a SNR of ∼ 17 dB SPL, the active noise canceling system was not 100% stable in that the dampened background could fluctuate; and we speculate that the effect of background noise interfered with performance in both participant groups on the ATD task. Nonetheless, the addition of background noise was not a problematic influence with respect to our most important finding–that our sample of aADHD were more susceptible to auditory distraction when task demands taxed cognitive resources. Third, care must be taken when generalizing results. The manifestation of ADHD in adults is highly heterogenous and often confounded by comorbidities. Around half of our sample were on stable SSRI medication which is indicative of earlier problems with anxiety and depression however, both anxiety and depression reside at the lower spectrum of expected of psychiatric comorbidities in adults with ADHD (Katzman et al., 2017). Our sample was also imbalanced in male:female ratio, of which there were more females than males with ADHD. Although childhood ADHD is more common in boys, differences in prevalence between sexes diminish almost completely in adulthood (Faraone et al., 2015; Matte et al., 2015) and symptomatic differences in hyperactivity and inattention between sexes also wane with older age (Ramtekkar et al., 2010); so we should not expect the gender imbalance in our sample to dramatically affect more general conclusions of our results. That notwithstanding, our sample was small and relatively high-functioning and may not be entirely representative for the more severe spectrum of the disorder. If, however subjects with ADHD in general are expected to have more severe symptoms and functional impairment, group differences detected between our sample and controls are likely to underestimate the overall differences rendering our results conservative. With respect to these limitations, we have cautiously opted to confine discussion to our small sample rather than boldly extrapolate our findings to the population as a whole. That being said, our general findings contribute important evidence to extant theories of cognitive control, auditory distraction, and the pathophysiology of ADHD in adults.

## Conclusion

To conclude, our sample of relatively high functioning young adults with ADHD were able to modulate auditory and SN systems in response to noise interference and perform at a commensurate level to controls when WM task demands were low. But when WM task demands were high, ADHD participants had difficulties attenuating task-irrelevant auditory cortical processing. Heightened auditory activity to task-irrelevant sound was associated with both poorer task performance and symptomatic inattentiveness. Our findings contribute to developmental models of persistent ADHD and more generally, WM capacity models of distraction; and demonstrate that an individual’s ability to regulate attentional engagement and impede auditory distraction is intricately related to their capacity for cognitive control. The study also has important clinical implications for aADHD underscoring the need for early interventions to adapt study/work environments, develop effective coping strategies, and minimize risk for chronic stress and anxiety in this vulnerable group.

## Data Availability Statement

The raw data supporting the conclusions of this article will be made available by the authors, without undue reservation.

## Ethics Statement

The studies involving human participants were reviewed and approved by the Regional Ethical Review Board in Linköping, Sweden (Dnr 2019-06158). The patients/participants provided their written informed consent to participate in this study.

## Author Contributions

JR, RB, AJC, and HD contributed to the conception and design of the study. AJC and RB were responsible for participant recruitment and data collection. RB was responsibility for analysis and manuscript drafting under the supervision of CS, HD, and JR. All authors scrutinized the statistical analysis, contributed to the manuscript’s revision and approved submitted version.

## Conflict of Interest

The authors declare that the research was conducted in the absence of any commercial or financial relationships that could be construed as a potential conflict of interest.

## Publisher’s Note

All claims expressed in this article are solely those of the authors and do not necessarily represent those of their affiliated organizations, or those of the publisher, the editors and the reviewers. Any product that may be evaluated in this article, or claim that may be made by its manufacturer, is not guaranteed or endorsed by the publisher.

## Footnotes

1
www.neurobs.com

2
www.nitrc.org/projects/conn

3
https://www.nitrc.org/projects/rex
