# Supplementary material for: The Effects of Working Memory Load on Auditory Distraction in Adults With Attention Deficit Hyperactivity Disorder
Source: Front Hum Neurosci. 2021 Nov 30;15:771711. doi: 10.3389/fnhum.2021.771711 (PMC8670091; doi:10.3389/fnhum.2021.771711)
Supplement: Supplementary file 1 [file Data_Sheet_1.docx]

Supplementary Material

Table S1

Cluster-level statistics for whole-brain within-group analyses. Reported *p*-values are FWE-corrected; and peak *x*, *y*, *z* coordinates correspond to MNI space. *k* = cluster size; *ns* = no significant clusters; L: left; R: right; SMG: supramarginal gyrus; SMA: supplementary motor area; STG: superior temporal gyrus; IOG: inferior occipital gyrus; ITG: inferior temporal gyrus; SPL: superior parietal lobe; MFG: middle frontal gyrus; Ext: exterior; OFuG: occipital fusiform gyrus.

| Contrast |  | Group |  | Brain Region |  | *k* |  | *p* |  | *x y z* |
| --- | --- | --- | --- | --- | --- | --- | --- | --- | --- | --- |
| ATD–rest |  | ADHD |  | SMG right |  | 47 |  | .000 |  | 54 -40 46 |
|  |  |  |  | Planum temporale R |  | 48 |  | .000 |  | 66 -24 12 |
|  |  |  |  | Planum temporale L |  | 126 |  | .000 |  | -58 -38 14 |
|  |  | Controls |  | Heschl’s gyrus L |  | 862 |  | .000 |  | -50 -24 8 |
|  |  |  |  | SMA |  | 173 |  | .000 |  | 0 6 56 |
|  |  |  |  | STG R |  | 496 |  | .000 |  | 60 -20 4 |
|  |  |  |  |  |  | 132 |  | .000 |  | 66 -40 20 |
|  |  |  |  | Planum polare L |  | 127 |  | .000 |  | -50 -8 -4 |
|  |  |  |  | MFG R |  | 17 |  | .000 |  | 32 38 28 |
|  |  |  |  | Anterior insula L |  | 54 |  | .000 |  | -36 16 4 |
| 0-back–ATD |  | ADHD |  | IOG L |  | 29 |  | .000 |  | -36 -82 2 |
|  |  |  |  | ITG L |  | 10 |  | .001 |  | -46 -52 -14 |
|  |  | Controls |  | Ext. cerebellum R |  | 19 |  | .000 |  | 4 -72 -28 |
| 1-back–ATD |  | ADHD |  | SPL L |  | 49 |  | .000 |  | -30 -48 34 |
|  |  |  |  | MFG L |  | 35 |  | .000 |  | -44 14 30 |
|  |  | Controls |  | SPL R |  | 352 |  | .000 |  | 38 -44 46 |
|  |  |  |  | Ext. cerebellum R |  | 39 |  | .000 |  | 28 -70 -50 |
|  |  |  |  |  |  | 10 |  | .001 |  | 8 -74 -24 |
|  |  |  |  | SPL L |  | 353 |  | .000 |  | -30 -62 52 |
|  |  |  |  | Fusiform gyrus L |  | 46 |  | .000 |  | -38 -62 -12 |
|  |  |  |  | Ext. cerebellum L |  | 10 |  | .001 |  | -6 -78 -26 |
| 2-back–ATD |  | ADHD |  | Ext. cerebellum L |  | 21 |  | .000 |  | -18 -58 -36 |
|  |  |  |  | Thalamus L |  | 112 |  | .000 |  | -12 -2 12 |
|  |  |  |  | SMG L |  | 90 |  | .000 |  | -40 -46 44 |
|  |  |  |  | Ext. cerebellum R |  | 19 |  | .000 |  | 10 -80 -38 |
|  |  |  |  | Putamen L |  | 30 |  | .000 |  | -26 16 0 |
|  |  |  |  | ITG L |  | 19 |  | .000 |  | -46 -56 -14 |
|  |  |  |  | SPL R |  | 20 |  | .000 |  | 30 -60 50 |
|  |  |  |  |  |  | 15 |  | .001 |  | 32 -50 44 |
|  |  |  |  | SPL L |  | 27 |  | .000 |  | -24 -64 42 |
|  |  | Controls |  | SPL L |  | 1255 |  | .000 |  | -18 -64 48 |
|  |  |  |  | OFuG L |  | 190 |  | .000 |  | -38 -66 12 |
|  |  |  |  | Ext. cerebellum R |  | 341 |  | .000 |  | 8 -78 -26 |
|  |  |  |  |  |  | 85 |  | .000 |  | 34 -70 -50 |
|  |  |  |  |  |  | 140 |  | .000 |  | 28 -60 -32 |
|  |  |  |  | SPL R |  | 641 |  | .000 |  | 38 -44 48 |
|  |  |  |  | MFG L |  | 68 |  | .000 |  | -52 20 36 |
|  |  |  |  |  |  | 136 |  | .000 |  | -28 0 56 |
|  |  |  |  | SMA L |  | 75 |  | .000 |  | -8 16 44 |
|  |  |  |  | Ext. cerebellum L |  | 23 |  | .000 |  | -36 -66 -48 |
|  |  |  |  | MFG R |  | 15 |  | .001 |  | 40 28 32 |
|  |  |  |  |  |  | 11 |  | .002 |  | 32 10 58 |
|  |  |  |  | Anterior insula L |  | 20 |  | .000 |  | -32 18 -6 |

Figure S1

Brain areas showing significant activation for the ATD–Rest contrast (ATD = auditory target detection). Left panel: ADHD (height threshold: *t* = 7.2, *p*-FWE = .05), right panel: Controls (height threshold: *t* = 7.6, *p*-FWE = .05).


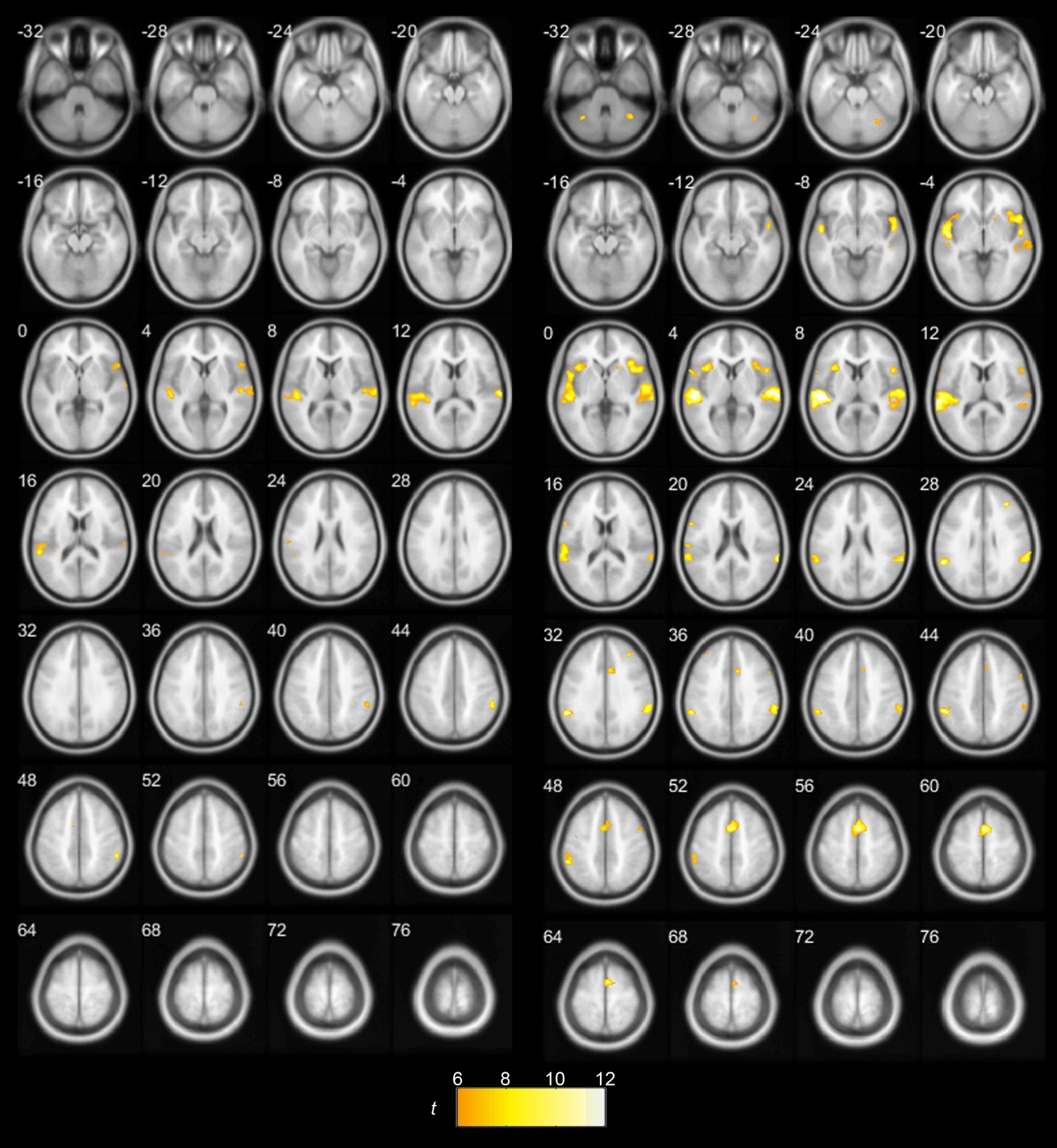


Figure S2

Brain areas showing significant activation for the 0-back–ATD contrast (ATD = auditory target detection). Left panel: ADHD (height threshold: *t* = 7.4, *p*-FWE = .05), right panel: Controls (height threshold: *t* = 7.8, *p*-FWE = .05).


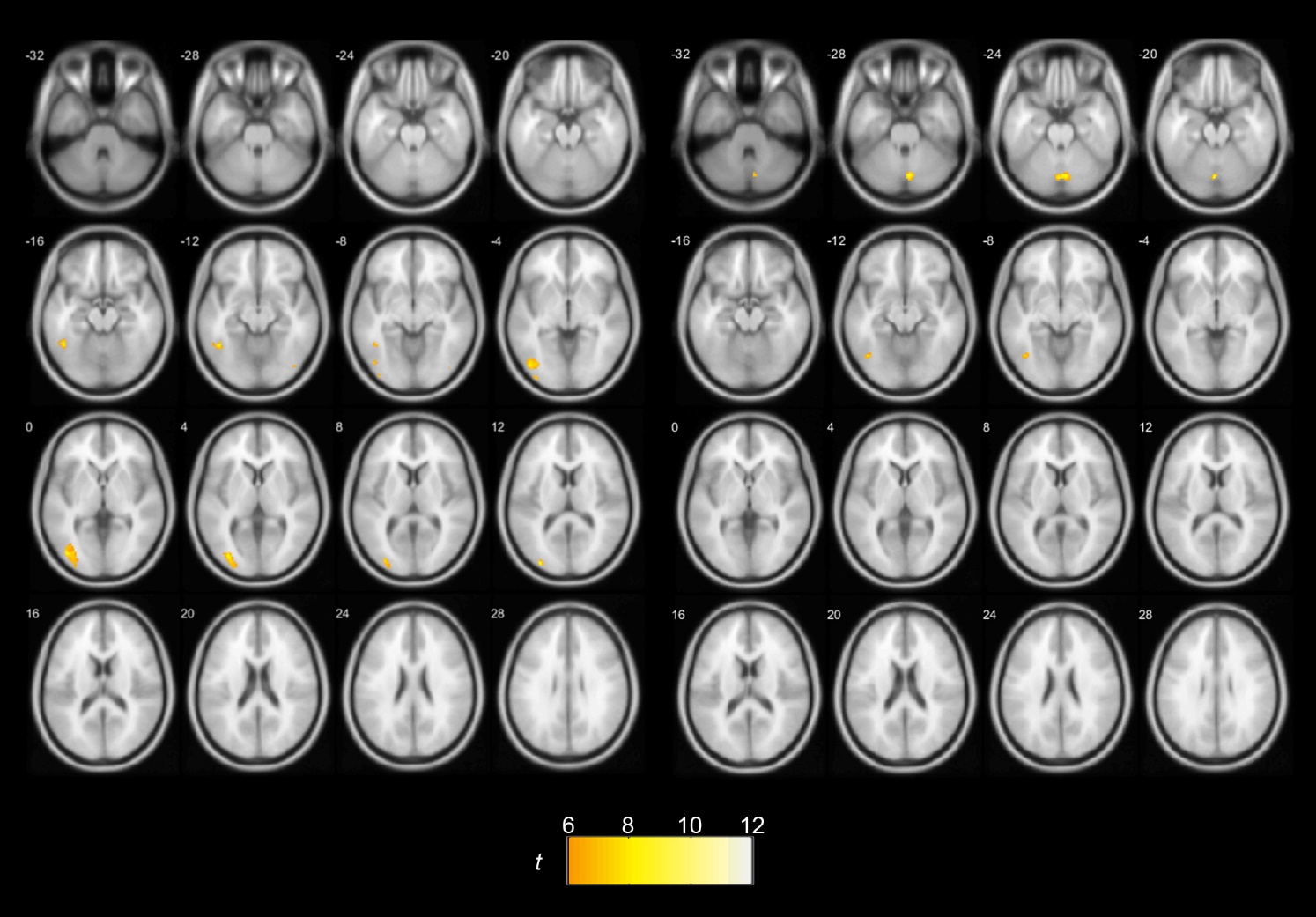


Figure S3

Brain areas showing significant activation for the 1-back–ATD contrast (ATD = auditory target detection). Left panel: ADHD (height threshold: t = 7.5, p-FWE = .05), right panel: Controls (height threshold: *t* = 7.7, *p*-FWE = .05).


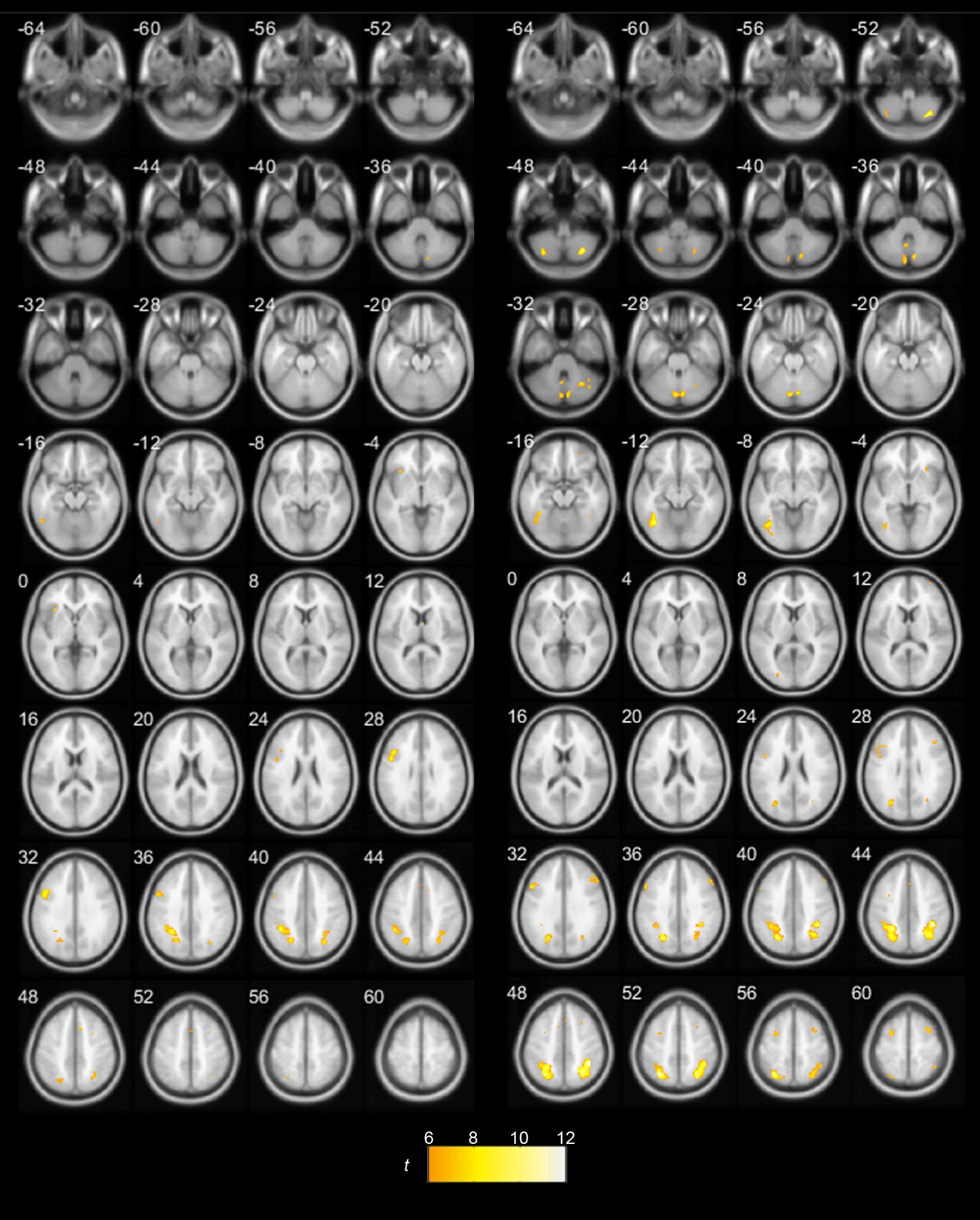


Figure S4

Brain areas showing significant activation for the 2-back–ATD contrast (ATD = auditory target detection). Left panel: ADHD (height threshold: *t* = 7.6, *p*-FWE = .05), right panel: Controls (height threshold: *t* = 7.5, *p*-FWE = .05).


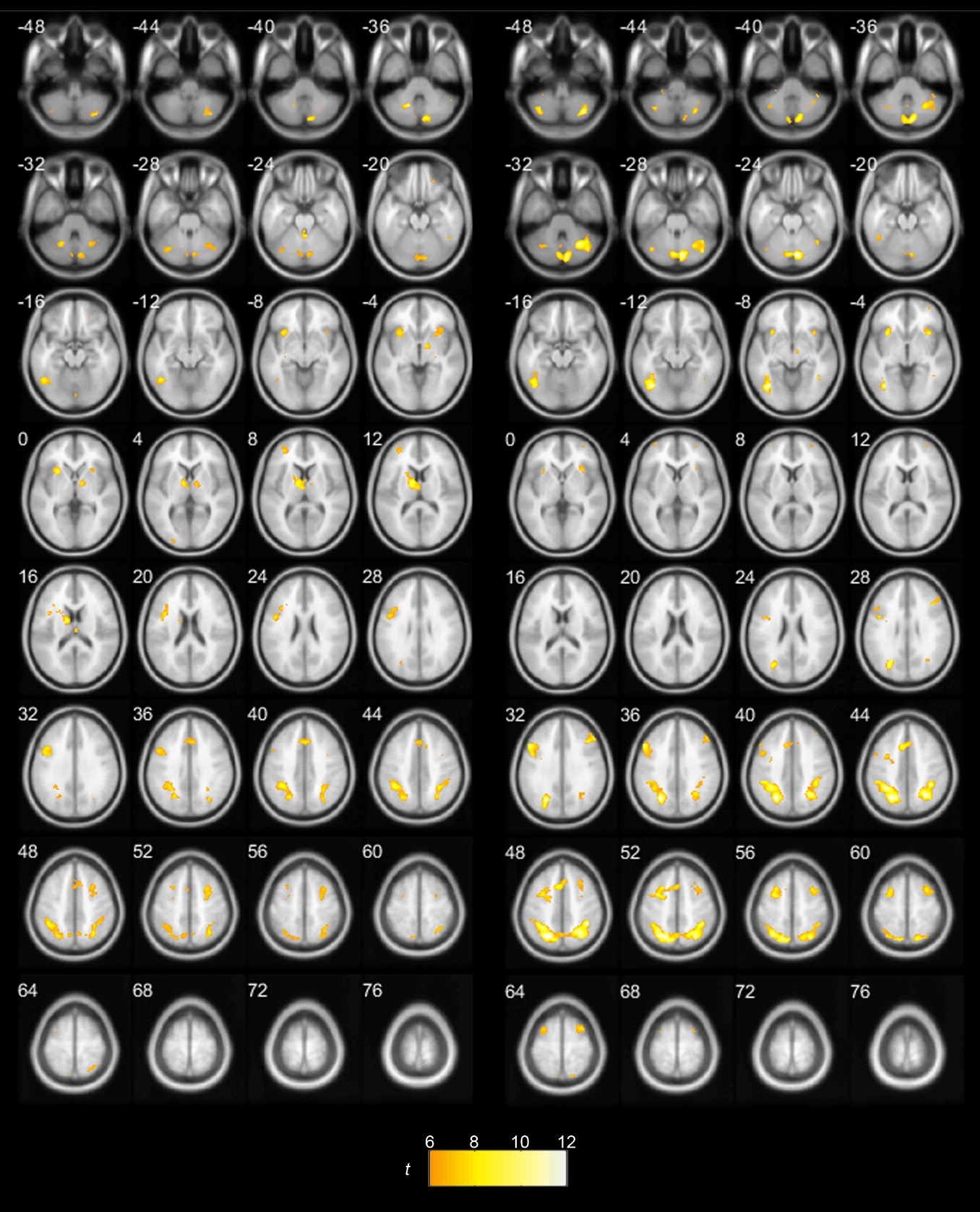


**Table S2**

Within-group analysis results for the auditory target detection contrast (ATD > rest). One-sample *t-*tests were used to determine if the mean BOLD activity within each ROI (per group) was greater than zero. Table shows for each group, the means (M), standard deviations (SD), *t-*scores and Bonferroni-corrected *p*-values per set of ROIs: left hemisphere auditory ROIs, right hemisphere auditory ROIs, and salience network hubs. L: left; R: right; dp: dysgranular posterior; gp: granular posterior; ACC: anterior cingulate cortex; PFC: prefrontal cortex.

| ROI | |  | Controls (*n* = 17) | | |  | ADHD (*n* = 17) | | |
| --- | --- | --- | --- | --- | --- | --- | --- | --- | --- |
| Auditory | |  | M (SD) | *t* (16) | *p* |  | M (SD) | *t* (16) | *p* |
| L | Heschl’s gyrus |  | 0.24 (0.2) | 10.3 | .000 |  | 0.47 (0.5) | 5.9 | .000 |
|  | planum polare |  | 0.35 (0.2) | 8.0 | .000 |  | 0.34 (0.4) | 3.7 | .005 |
|  | planum temporale |  | 0.59 (0.2) | 16.8 | .000 |  | 0.71 (0.4) | 7.3 | .000 |
|  | dp insula |  | 0.13 (0.2) | 2.1 | .118 |  | 0.23 (0.4) | 2.2 | .096 |
|  | gp insula |  | -0.01 (0.7) | -0.2 | .989 |  | 0.12 (0.4) | 1.5 | .349 |
| R | Heschl’s gyrus |  | 0.14 (0.2) | 4.2 | .002 |  | 0.36 (0.4) | 4.2 | .002 |
|  | planum polare |  | 0.25 (0.2) | 5.7 | .000 |  | 0.28 (0.4) | 3.0 | .022 |
|  | planum temporale |  | 0.38 (0.2) | 6.7 | .000 |  | 0.47 (0.4) | 5.8 | .000 |
|  | dp insula |  | -0.07 (0.2) | -1.6 | .999 |  | 0.03 (0.3) | 0.5 | .862 |
|  | gp insula |  | -0.14 (0.1) | -4.1 | 1.00 |  | -0.04 (0.3) | -0.4 | .999 |
| Salience network | |  | M (SD) | *t* (16) | *p* |  | M (SD) | *t* (16) | *p* |
| dorsal ACC | |  | 0.24 (0.2) | 4.3 | .002 |  | 0.19 (0.4) | 2.5 | .085 |
| L | anterior insula |  | 0.39 (0.2) | 7.5 | .000 |  | 0.34 (0.4) | 4.0 | .003 |
|  | rostral PFC |  | 0.21 (0.2) | 3.2 | .018 |  | 0.23 (0.4) | 1.7 | .302 |
|  | supramarginal gyrus |  | 0.31 (0.2) | 7.3 | .000 |  | 0.26 (0.2) | 4.5 | .001 |
| R | anterior insula |  | 0.5 (0.2) | 8.7 | .000 |  | 0.55 (0.4) | 5.7 | .000 |
|  | rostral PFC |  | 0.23 (0.2) | 4.5 | .001 |  | 0.32 (0.4) | 4.0 | .003 |
|  | supramarginal gyrus |  | 0.29 (0.2) | 7.7 | .000 |  | 0.24 (0.3) | 4.2 | .002 |

**Table S3**

Post hoc analysis results exploring the effect of working memory (WM) load on auditory attenuation. Table shows for each group, the estimated marginal means (M), standard errors (SE), *F-*scores and Bonferroni-corrected *p*-values per interaction of interest: WM load × Group per left (L) and right (R) hemisphere; as well as High load (2-back) × ROI × Group per hemisphere. dp: dysgranular posterior; gp: granular posterior.

| Interaction | |  | M (SE) | |  | Group comparison | |
| --- | --- | --- | --- | --- | --- | --- | --- |
| WM load × Group | |  | Controls | ADHD |  | *F* (1, 32) | *p* |
| L | 0-back |  | -.04 (.06) | -.14 (.06) |  | 1.3 | .262 |
|  | 1-back |  | -.23 (.05) | -.22 (.05) |  | 0.0 | .871 |
|  | 2-back |  | -.50 (.06) | -.29 (.06) |  | 6.2 | .018 |
| R | 0-back |  | -.04 (.06) | -.08 (.06) |  | 0.3 | .566 |
|  | 1-back |  | -.21 (.05) | -.18 (.05) |  | 0.1 | .750 |
|  | 2-back |  | -.44 (.06) | -.24 (.06) |  | 6.1 | .019 |
| High load × ROI × Group | |  | Controls | ADHD |  | *F* (1, 32) | *p* |
| L | Heschl’s gyrus |  | -.44 (.07) | -.30 (.07) |  | 1.9 | .176 |
|  | planum polare |  | -.70 (.06) | -.32 (.06) |  | 18.7 | .000 |
|  | planum temporale |  | -.50 (.08) | -.34 (.08) |  | 1.9 | .173 |
|  | dp insula |  | -.45 (.05) | -.26 (.05) |  | 6.4 | .017 |
|  | gp insula |  | -.39 (.06) | -.23 (.06) |  | 3.8 | .059 |
| R | Heschl’s gyrus |  | -.43 (.07) | -.25 (.07) |  | 3.3 | .079 |
|  | planum polare |  | -.60 (.08) | -.28 (.08) |  | 8.5 | .006 |
|  | planum temporale |  | -.55 (.08) | -.34 (.08) |  | 3.8 | .059 |
|  | dp insula |  | -.30 (.04) | -.14 (.04) |  | 6.3 | .017 |
|  | gp insula |  | -.33 (.06) | -.19 (.06) |  | 3.0 | .093 |
